# Peripheral Vestibular Dysfunction Is a Common Occurrence in Children With Non-syndromic and Syndromic Genetic Hearing Loss

**DOI:** 10.3389/fneur.2021.714543

**Published:** 2021-10-21

**Authors:** Alicia Wang, A. Eliot Shearer, Guang Wei Zhou, Margaret Kenna, Dennis Poe, Greg R. Licameli, Jacob R. Brodsky

**Affiliations:** ^1^Department of Otolaryngology and Communication Enhancement, Boston Children's Hospital, Boston, MA, United States; ^2^Department of Otolaryngology, Harvard Medical School, Boston, MA, United States

**Keywords:** genetic hearing loss, peripheral vestibular disorder, syndromic hearing loss, vestibular loss, vestibular testing, cochlear implant, pediatric vestibular, congenital hearing loss

## Abstract

Hearing loss (HL) is the most common sensory deficit in humans and is frequently accompanied by peripheral vestibular loss (PVL). While often overlooked, PVL is an important sensory dysfunction that may impair development of motor milestones in children and can have a significant negative impact on quality of life. In addition, many animal and *in vitro* models of deafness use vestibular hair cells as a proxy to study cochlear hair cells. The extent of vestibular end organ dysfunction associated with genetic pediatric hearing loss is not well-understood. We studied children with a known genetic cause of hearing loss who underwent routine preoperative vestibular testing prior to cochlear implantation between June 2014 and July 2020. Vestibular testing included videonystagmography, rotary chair, video head impulse testing, and/or vestibular evoked myogenic potentials. Etiology of HL was determined through history, physical examination, imaging, laboratory testing, and/or genetic testing. Forty-four children (21 female/23 male) met inclusion criteria; 24 had genetic non-syndromic and 20 had genetic syndromic forms of HL. Mean age at the time of testing was 2.8 ± 3.8 years (range 7 months−17 years). The most common cause of non-syndromic HL was due to mutations in GJB2 (*n* = 13) followed by MYO15A (3), MYO6 (2), POU3F4 (2), TMPRSS3 (1), CDH23 (1), TMC1 (1), and ESRRB (1). The most common forms of syndromic HL were Usher syndrome (4) and Waardenburg (4), followed by SCID/reticular dysgenesis (3), CHARGE (2), CAPOS (1), Coffin-Siris (1), Jervell and Lange-Nielsen (1), Noonan (1), peroxisome biogenesis disorder (1), Perrault (1), and Trisomy 21 (1). Overall, 23 patients (52%) had PVL. A larger proportion of children with syndromic forms of HL had PVL (12/20, 60%) compared with children with genetic non-syndromic HL (11/24, 46%), though without statistical significant (*p* = 0.3). The occurrence of PVL varied by affected gene. In conclusion, PVL is a common finding in children with syndromic and non-syndromic genetic HL undergoing vestibular evaluation prior to cochlear implantation. Improved understanding of the molecular physiology of vestibular hair cell dysfunction is important for clinical care as well as research involving vestibular hair cells in model organisms and *in vitro* models.

## Introduction

Hearing loss is the most common sensory impairment in humans, affecting nearly 5% of the world population (1). In children, hearing loss may be acquired due to environmental damage from infections such as cytomegalovirus and meningitis, hypoxia, or hyperbilirubinemia (1). However, in developed countries, a genetic etiology is more likely, accounting for over 50% of childhood hearing loss cases (2). Genetic hearing loss may either be present at birth (i.e., congenital) or be identified and progress after the neonatal period. Hearing loss with genetic origins can appear either as an isolated finding (non-syndromic) or be associated with other disorders, such as vision, heart, or kidney abnormalities (syndromic). To date, there are 123 genes known to cause hearing loss not associated with other clinical features (termed “non-syndromic hearing loss”). In addition, there are several hundred known syndromic forms of hearing loss (e.g., Usher syndrome, CHARGE syndrome, and Pendred syndrome) (2).

Peripheral vestibular loss (PVL) frequently accompanies hearing loss, and vestibular impairment is estimated to affect around 50% of patients with sensorineural hearing loss (SNHL) (3–6). Such a high prevalence likely reflects the shared anatomy and physiology between the vestibular organs (labyrinth) and auditory organ (cochlea). The cochlea and vestibule are a single complex interdependent system closely tied in embryologic development that share the same extracellular fluids, transmit information *via* the same nerve (CN VIII, vestibulocochlear nerve), and contain paralogous structures that are responsible for mechanotransduction of stimuli (hair cells). As such, it follows that pathology that causes hearing loss can also frequently cause PVL.

Although the genetic basis of hearing loss has been studied extensively over the past three decades, the genetic basis of PVL merits further study. While there are well-known associations between vestibular impairment and specific syndromic forms of hearing loss—for example, Usher syndrome and Waardenburg syndrome—the correlation between the known genotype and vestibular phenotype remains poorly defined (7–9). Published studies of peripheral vestibular loss in the setting of non-syndromic genetic hearing loss particularly sparse, although there has been a limited number of investigations of vestibular function in patients with mutations in the *GJB2* gene (10). Isolated genetic PVL in the absence of hearing loss is especially uncommon and even more poorly described (11, 12).

The presence of PVL in the setting of genetic hearing loss is of clinical importance. Children with hearing loss are at significant risk for poor speech, hearing, and social development (13). Dysfunction of the vestibular end organs in these children can lead to additional morbidity, including poor motor development and balance deficits (14, 15). These factors all contribute to a reduced quality of life. Children undergoing evaluation for cochlear implantation are affected by severe-to-profound hearing loss that is not effectively treated by hearing aids. Given this primary sensory deficit, a major clinical goal is to assess and treat any other form of reduced sensory input, including vestibular impairment. It has been shown that gross motor delays and postural control impairments in this population can be significantly improved through vestibular rehabilitation; therefore, early identification of vestibular dysfunction is vital to achieving this goal (16). Children with SNHL who undergo cochlear implantation are also at increased risk of injury to the vestibular organs, with reported rates ranging from 20 to 80% (5, 6, 17–19). This further highlights the importance of early identification of vestibular dysfunction. An improved understanding of the relationship between genetic hearing loss and PVL will improve patient and family counseling regarding prognosis, cochlear implant surgery planning, and expectations for treatment. It is also of scientific interest to better characterize the molecular pathophysiology of vestibular loss, as many model organisms and *in vitro* models of deafness use vestibular hair cells as a proxy to study cochlear hair cells (20).

Our goal in this study was to investigate the prevalence of PVL prior to cochlear implantation in a cohort of children with a known genetic hearing loss. Specific outcome measures included vestibular testing results and results from standardized motor evaluations. We demonstrate a relatively high prevalence of PVL in children with both syndromic and non-syndromic forms of genetic hearing loss that is genotype dependent. This study has implications for evaluation and habilitation of children with hearing loss.

## Materials and Methods

### Subjects

We conducted a retrospective review of patients <18 years of age with SNHL who met the following criteria: (1) an identified genetic cause of hearing loss, and (2) completion of vestibular testing prior to cochlear implantation at our institution between June 2014 and July 2020. Patients were identified through our internal REDCap database of patients who have undergone cochlear implantation at our institution (21, 22). Further review of the medical record was performed for patients meeting inclusion criteria to evaluate specific characteristics, including etiology of hearing loss, results of vestibular testing, and imaging findings. This study was approved by the Institutional Review Board at our hospital.

### Clinical Evaluation

Hearing loss etiology was determined through history, physical examination, imaging, laboratory testing, and clinical genetic testing. Genetic testing was not standardized and was performed on a case-by-case basis based on clinical judgement and test availability. Genetic tests performed included single-gene sequencing (i.e., *GJB2*), multi-gene panels including dozens to hundreds of genes sequenced, and exome sequencing. Test interpretation was performed in accordance with consultation from a geneticist, when necessary.

### Vestibular Evaluation

Vestibular testing was performed by a licensed audiologist with the support of a trained assistant according to a standardized protocol that we have previously described in greater detail (23). Rotary chair testing was conducted using the Micromedical System 2000 (Micromedical Technologies, Chatham, Illinois). In older patients, rotary chair testing was conducted using videonystagmography (VNG) goggles. For patients ≤ 3 years of age, an infrared camera was used with the child seated on a parent's lap in the darkened rotary chair enclosure. Cervical and ocular vestibular evoked myogenic potential testing (cVEMP and oVEMP) were recorded using the Bio-logic Navigator Pro Evoked Potential system (Natus Medical). Video head impulse testing (VHIT) was conducted using the ICS Impulse system (GN Otometrics, Taastrup, Denmark).

When conducted using VNG goggles, rotary chair results were considered abnormal if gains for at least three frequencies tested were below the age-adjusted normal range and phase leads were elevated above the age-adjusted normal range for at least three frequencies, or the time constant was <12 s. When conducted with the observational camera technique, results were considered abnormal if either a significant asymmetry was detected between clockwise and counterclockwise rotation or the vestibulo-ocular reflex was absent with rotation in at least one direction. VEMP testing was considered abnormal if responses were absent in the tested ear at all stimulus thresholds. VHIT testing was considered abnormal if the vestibulo-ocular reflex gain was below 0.7 and corrective saccades were observed for at least one semicircular canal plane. Patients were considered to have evidence of peripheral vestibular loss (PVL) if results of at least one vestibular test were abnormal.

Toys with flashing lights were used to attract young childrens' attention during testing, when needed, including to control direction of gaze during VNG testing and to maintain appropriate neck turning and tension during cVEMP testing. Continuous electromyography was monitored during cVEMP and oVEMP testing to ensure continued sufficient muscle contraction throughout the test. The acquisition software automatically rejected any VEMP response with inadequate muscle tension.

### Motor Evaluation

Many patients also underwent routine motor evaluation by a licensed physical therapist prior to cochlear implantation. Patients <5 years of age were assessed using the Peabody Developmental Motor Scales, Second Edition (PDMS-2) Gross Motor Quotient (GMQ) (24). The GMQ is derived from scores on three of four subtests: reflexes (8 items), stationary (30 items), locomotion (89 items), and object manipulation (24 items). The total raw score on each subtest is converted to a standard score (ranging from 0 to 20) based on the subject's age and provided population normative data. The standard score is then summed and compared to normative data to determine the GMQ and GMQ percentile. Higher score values correspond to higher motor proficiency, and percentile ranks below 25% are suggested to be “below average” performance.

Patients ≥5 years of age were assessed using the Bruininks-Oseretsky Test of Motor Proficiency, Second Edition (BOT-2) body coordination composite score. This is derived from the BOT-2 bilateral coordination subtest and balance subtest, which consist of 7 and 9 items, respectively (25). The total point score on each subtest (ranging from 0 to 24 for bilateral coordination and 0 to 37 points for balance) is converted to a scale score based on the subject's age, sex, and provided population-level normative data. The two scale scores are summed to produce the body coordination score, which is converted to a standard score and percentile rank, again based on the subject's age, sex, and comparison to normative data. Higher score values correspond to higher motor proficiency, and percentile ranks below 18% are suggested to be “below average” performance. Both the GMQ and BOT-2 body coordination scores were converted to percentile ranks for analysis.

### Statistical Methods

Statistical analyses were conducted using R version 3.6.3 (R Core Team, Vienna, Austria). Differences in characteristics between patient groups were assessed using a chi-square test for categorical variables. An independent-samples *t-*test was used to assess differences in motor function outcomes between patient groups. A Mann–Whitney *U*-test was used to assess differences in age between patient groups.

## Results

### Demographics

We initially identified 149 patients who underwent routine pre-cochlear implant vestibular testing at our institution during the study timeframe. Of these, 44 patients had a known genetic cause of their hearing loss and had not previously undergone cochlear implantation. Demographics and clinical features of the study sample are summarized in Table 1. At the time of vestibular evaluation, patients ranged in age from 7.7 months to 17.5 years with a mean age of 2.8 years (SD 3.8). Most patients (38/44) were <5 years of age. Twenty-one patients (48%) were female and twenty-three (52%) were male.

**Table 1:** Clinical characteristics of patients with genetic hearing loss undergoing pre-cochlear implantation vestibular testing (*n* = 44).

**Clinical characteristics**	
Mean age ± SD, years	2.8 ± 3.8
Age range	7.7 months−17.5 years
Female, *n*	21
**Hearing loss etiology**, ***n***	
Genetic non-syndromic	24
Syndromic	20
**Hearing loss type**, ***n***	
Congenital profound, bilateral	32
Congenital progressive, bilateral	9
ANSD, bilateral	2
Asymmetric hearing loss	1
**CT/MRI findings**, ***n***	
Normal	37
Cochlea malformation	3
Cochleo-vestibular malformation	4

*ANSD, auditory neuropathy spectrum disorder*.

### Clinical Evaluation and Genetic Testing Results

As shown in Table 1, hearing loss was congenital and bilateral in 41/44 (93%) patients, with 32/44 (73%) bilaterally profound at birth, and 9/44 (20%) progressive. The remaining 3/44 patients had auditory neuropathy spectrum disorder (ANSD, 2/44, 5%) or asymmetric hearing loss (1/44, 2%). Genetic non-syndromic hearing loss was diagnosed in 24/44 (55%) subjects, while the remainder, 20/44 (45%), were diagnosed with syndromic hearing loss (Table 1). The most common cause of non-syndromic hearing loss was due to mutations in *GJB2* (*n* = 13) followed by *MYO15A* (3), *MYO6* (2), *POU3F4* (2), *TMPRSS3* (1), *CDH23* (1), *TMC1* (1), and *ESRRB* (1). The most common forms of syndromic hearing loss were Usher syndrome (4) and Waardenburg (4), followed by SCID/reticular dysgenesis (3), CHARGE (2), CAPOS (1), Coffin-Siris (1), Jervell and Lange-Nielsen (1), Noonan (1), peroxisome biogenesis disorder (1), Perrault (1), and Trisomy 21 (1). A full summary of causative genetic variants identified in the patient sample can be found in the Supplementary Material.

All patients had at least one imaging study performed. These included magnetic resonance imaging (MRI) of the temporal bones in 39/44 (89%) subjects and fine cut non-contrast computed tomography (CT) of the temporal bones in 39/44 (89%). Both forms of imaging were obtained in 34/44 (77%) subjects. No imaging abnormalities were identified in 37/44 (84%) subjects, a cochlear abnormality was identified in 3/44 (7%), and a cochleo-vestibular abnormality was identified in 4/44 (9%) (Table 1).

### Vestibular Testing and Gross Motor Function

All patients underwent cVEMP testing as part of their vestibular evaluation, and 43/44 (98%) also underwent rotary chair testing. Additionally, VHIT was performed in 6/44 (14%) patients and oVEMP testing in 7/44 (16%). Patients who underwent VHIT testing ranged from 5.0 to 17.5 years of age. Patients who underwent oVEMP testing ranged from 2.5 to 17.5 years of age. No patients underwent caloric testing.

Results of these specific vestibular tests are summarized in Table 2. Overall, 23 patients (52.3%) had evidence of peripheral vestibular loss (PVL), and 21/23 had bilateral deficits. Two patients had evidence of a unilateral deficit affecting only the otolith organs (Table 3). A greater proportion of patients with syndromic forms of hearing loss had PVL (12/20, 60.0%) compared to patients with non-syndromic hearing loss (11/24, 45.8%), although this difference was not statistically significant (*p* = 0.3). There was not a significant difference in age between patients with syndromic hearing loss (median = 11.6 months) compared to those with non-syndromic hearing loss (median = 16.2 months; *p* = 0.7). Patients with syndromic hearing loss were significantly more likely (*p* = 0.02) to have abnormal cVEMP outcomes (11/20, 55.0%) than patients with non-syndromic hearing loss (5/24, 20.8%).

**Table 2:** Results of vestibular testing in all patients with genetic hearing loss (*n* = 44).

**Hearing loss etiology**	**Rotary chair outcome**	**VHIT outcome**	**cVEMP outcome**	**oVEMP outcome**	**Overall vestibular function**
	**Abnormal/total (%)**	**Abnormal/total (%)**	**Abnormal/total (%)**	**Abnormal/total (%)**	**PVL/total (%)**
**Genetic NSHL**	**8/23 (34.8%)**	**0/3 (0.0%)**	**5/24 (20.8%)**	**2/4 (50.0%)**	**11/24 (45.8%)**
*CDH23*	0/1	0/1	0/1	0/1	0/1
*ESRRB*	0/1		1/1		1/1
*GJB2*	2/12	0/1	1/13	0/1	3/13
*MYO15A*	1/3	0/1	1/3	1/1	2/3
*MYO6*	2/2		1/2		2/2
*POU3F4*	2/2		1/2	1/1	2/2
*TMC1*	1/1		0/1		1/1
*TMPRSS3*	0/1		0/1		0/1
**Syndromic HL**	**10/20 (50.0%)**	**2/3 (66.7%)**	**11/20 (55.0%)**	**2/3 (66.7%)**	**12/20 (60.0%)**
CAPOS	1/1	1/1	1/1	1/1	1/1
CHARGE	2/2		2/2		2/2
Coffin-Siris	0/1		0/1		0/1
JLNS	1/1		1/1		1/1
Noonan	0/1		0/1		0/1
PBD	0/1		1/1	1/1	1/1
Perrault	1/1		1/1		1/1
SCID/RD	1/3		0/3		1/3
Trisomy 21	0/1		1/1		1/1
Usher	3/4	1/2	3/4	0/1	3/4
WS1	0/1		0/1		0/1
WS2	1/3		1/3		1/3
**Grand Total**	**18/43 (41.9%)**	**2/6 (33.3%)**	**16/44 (36.4%)**	**4/7 (57.1%)**	**23/44 (52.3%)**

*cVEMP, cervical vestibular evoked myogenic potential; oVEMP, ocular vestibular evoked myogenic potential; VHIT, video head impulse test; PVL, peripheral vestibular loss; JLNS, Jervell and Lange-Nielsen syndrome; PBD, peroxisome biogenesis disorder; SCID/RD, severe combined immunodeficiency/reticular dysgenesis; T21, trisomy 21; WS1, Waardenburg syndrome type 1; WS2, Waardenburg syndrome type 2*.

**Table 3:** Laterality of otolith and semicircular canal deficits among all patients (*n* = 44).

**Otolith deficit**	**Semicircular canal deficit**	
	***n*** **(% of column)**	
	**Bilateral**	**None**
Bilateral	11 (61.1%)	3 (11.5%)
Unilateral	2 (11.1%)	2 (7.7%)
None	5 (27.8%)	21 (80.8%)

**Figure 1:**
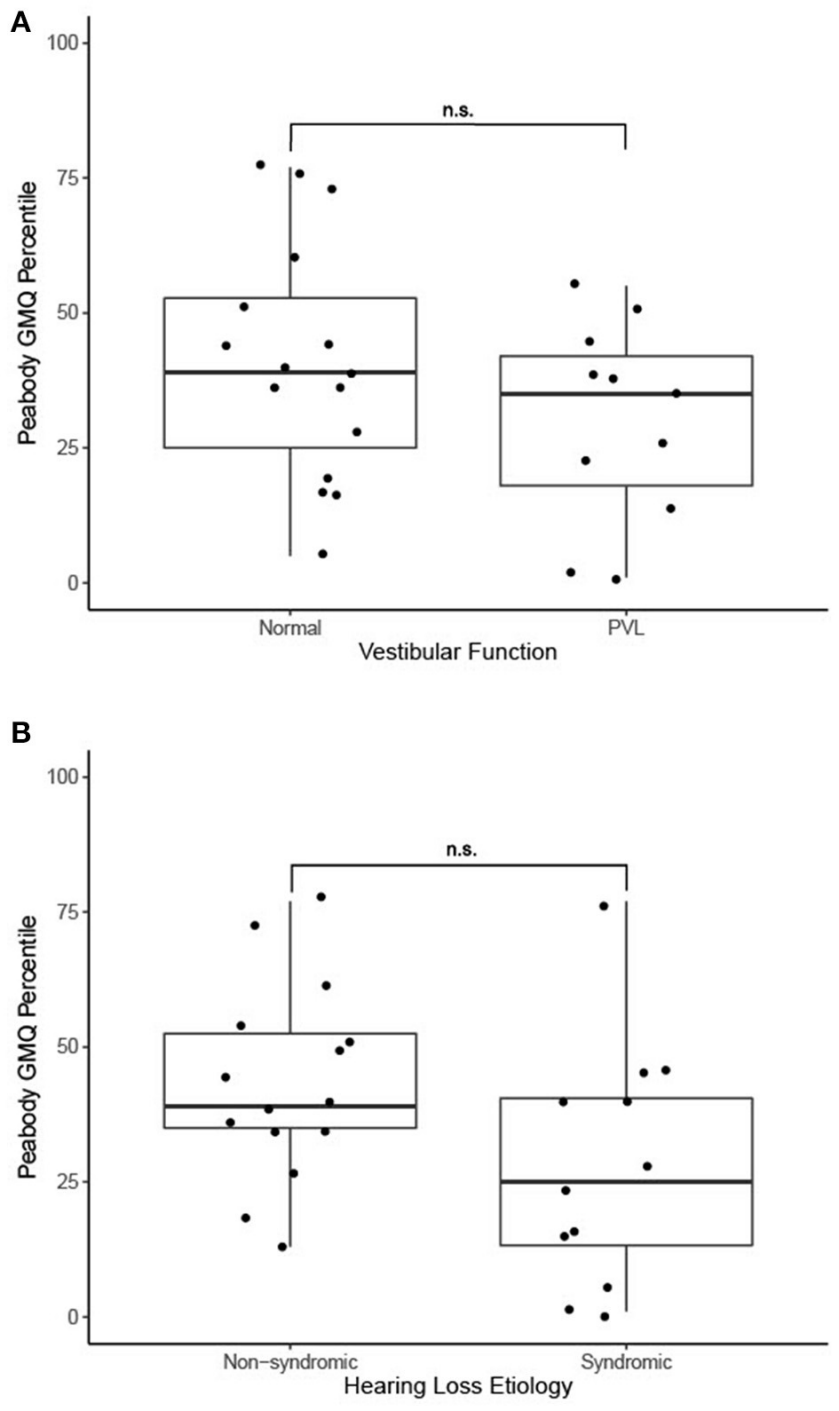
**(A)** Motor function outcomes in 27 patients with PVL and with normal vestibular function, n.s., no statistically significant difference (independent samples *t*-test). **(B)** Motor function outcomes in 27 patients with genetic non-syndromic and syndromic forms of hearing loss, n.s., no statistically significant difference (independent samples *t*-test).

Thirty-two patients underwent a gross motor evaluation by a physical therapist in addition to vestibular evaluation. The vast majority (*n* = 27, 84%) were evaluated using the PDMS-2 while the remainder were evaluated using the BOT-2. Among patients evaluated with the PDMS-2, the mean gross motor quotient (GMQ) percentile rank was 36.6 (SD 21.3). Among the five patients evaluated with the BOT-2, the mean body coordination percentile rank was 39.4 (SD 33.7). Patients with normal vestibular testing findings had a greater Peabody GMQ percentile rank score (*n* = 16, mean: 41.2 ± 22.3) compared to patients with evidence of PVL (*n* = 11, mean: 29.8 ± 18.6), though this difference did not reach statistical significance [*t*_(25)_ = 1.4, *p* = 0.2] (Figure 1A). Patients with syndromic hearing loss had a lower Peabody GMQ percentile rank score (*n* = 12, mean: 27.8 ± 22.4) compared to patients with non-syndromic hearing loss (*n* = 15, mean: 43.5 ± 18.1). This result also did not reach statistical significance [*t*_(25)_ = 2.0, *p* = 0.05] (Figure 1B).

## Discussion

Vestibular disorders lead to imbalance, postural instability, and visual difficulties with high-frequency movement, which can impair the development of gross motor skills in children (26). Additionally, vestibular impairment may add to developmental difficulties in children with hearing loss who are already affected by one or more other sensory disorders. However, the extent to which both the vestibular and cochlear sensory systems are simultaneously affected in children with hearing loss has only recently been studied. Cushing et al. found that 100% (11/11) of children with hearing loss due to meningitis were affected by PVL compared to 46% (14/31) of children with anatomic anomalies (3). In a recent study of 195 children with hearing loss and cochleo-vestibular malformations, 15.9% had measured vestibular impairment on testing and 21.6% showed delayed motor milestones (27). However, there is limited and conflicting data on the vestibular implications of non-syndromic mutations in the 120 known deafness-causing genes. One study showed 66% (11/24) of children with non-syndromic hearing loss due to mutations in *GJB2* were noted to have PVL (3). Conversely, another study noted a much smaller rate of PVL, 23% (3/13), in a similar group of children with *GJB2* mutations causing hearing loss (26).

One limitation of many studies of vestibular function in children with hearing loss is limited access to comprehensive vestibular testing in younger children. As a result, many such studies have been limited to only VEMP testing. In our study we noted that children with syndromic and non-syndromic hearing loss have a similar rate of PVL, but that cVEMP abnormalities were more common in children with syndromic hearing loss. Thus, studies of only cVEMP testing may miss evidence of PVL in many children with non-syndromic hearing loss. We only found abnormal cVEMP results in 1/13 patients with *GJB2* mutation, but 2/12 patients with *GJB2* mutations had abnormal rotary chair testing results, both of whom had normal cVEMP responses. Thus, the vestibular effects of *GJB2* may be variable and could be underestimated by studies that do not include tests of semicircular canal function. Although pediatric vestibular medicine is a relatively new field, the number of dedicated pediatric vestibular programs around the world has grown in recent years (28). This has been occurring concurrently with the rapid growth of affordable and comprehensive genetic testing capabilities for children with congenital hearing loss. Thus, our ability to study the vestibular implications of both syndromic and non-syndromic genetic hearing loss is expected to increase exponentially in the near future.

The goal of this study was to evaluate the extent of PVL in children with genetic causes of severe to profound hearing loss undergoing cochlear implantation. This is the largest study to date of children with a known genetic diagnosis who underwent a comprehensive objective testing of pre -operative vestibular function. Our data demonstrates that PVL commonly affects children with both syndromic and non-syndromic causes of genetic hearing loss in a genotype dependent manner. Overall, the rate of PVL was slightly higher in the syndromic vs. the non-syndromic group (60 and 49%, respectively). The results from this study expand the genotype-phenotype correlations of prior studies with respect to genetic mutation and vestibular dysfunction in the setting of genetic hearing loss. In particular, we show measurable effects on the peripheral vestibular system for mutations in six of eight causes of apparent genetic non-syndromic hearing loss. Further research should focus on expanded testing of the vestibular system in children and adults with hearing loss. This study also demonstrates trends toward delayed motor development in patients with PVL vs. those with normal vestibular function, as well as patients with syndromic forms of hearing loss. Our results further emphasize the importance of early identification and rehabilitation of vestibular deficits in patients with genetic hearing loss. Delayed motor development in patients with syndromic hearing loss may be multifactorial and result from dysfunction in systems other than or including the vestibular system.

A gene-specific understanding of the effect of mutations on the cochleovestibular system is necessary to improve pre-operative counseling for children with severe to profound hearing loss and to guide subsequent rehabilitation. Further insight into the effects of specific genetic mutations on PVL will also have important implications for future research on genetic hearing lossMany scientists use vestibular hair cells as a proxy for *in vitro* studies of human cochlear hair cell function (20, 29). This is due to the relative ease of harvest of the vestibular hair cells in animal models, the reproducibility of differentiation of stem cells in to vestibular hair cells, and the physiologic similarity of the vestibular hair cells to cochlear hair cells. And finally, in the context of gene therapy for deafness, an understanding of the effect of any therapy on the vestibular system will be crucial for optimal outcomes (30).

## Data Availability Statement

This study is a retrospective analysis of confidential clinical genetic analyses that were analyzed in an anonymized fashion for the purposes of this study. The raw data from the anonymized analyses are available from the corresponding author upon request. Requests to access these datasets should be directed to Jacob R. Brodsky, jacob.brodsky@childrens.harvard.edu.

## Author Contributions

AW, AS, GZ, MK, DP, GL, and JB: study concept, design, manuscript review, revision, and approval. AW, AS, MK, and JB: data collection and analysis and manuscript composition. All authors contributed to the article and approved the submitted version.

## Funding

All financial support for this study was provided by the Department of Otolaryngology and Communication Enhancement at Boston Children's Hospital.

## Conflict of Interest

The authors declare that the research was conducted in the absence of any commercial or financial relationships that could be construed as a potential conflict of interest.

## Publisher's Note

All claims expressed in this article are solely those of the authors and do not necessarily represent those of their affiliated organizations, or those of the publisher, the editors and the reviewers. Any product that may be evaluated in this article, or claim that may be made by its manufacturer, is not guaranteed or endorsed by the publisher.
