# Supplementary material for: Peripheral Vestibular Dysfunction Is a Common Occurrence in Children With Non-syndromic and Syndromic Genetic Hearing Loss
Source: Front Neurol. 2021 Oct 21;12:714543. doi: 10.3389/fneur.2021.714543 (PMC8567025; doi:10.3389/fneur.2021.714543)
Supplement: Supplementary file 1 [file Table_1.DOCX]

**Supplemental Table 1A: Causative mutations for non-syndromic hearing loss (n=24)**

| ID | Vestibular Function | Hearing Loss Etiology | Causative Genetic Variants | Zygosity | Inheritance |
| --- | --- | --- | --- | --- | --- |
| 882 | PVL | *ESRRB* | *ESRRB* c.745C>T (p.Arg249X) | Het | AR |
| 799 | Normal | *CDH23* | *CDH23* c.5237G>A (p.Arg1746Gln)  *CDH23* c.4879G>A (p.Glu1627Lys) | Het  Het | AR |
| 703 | Normal | *GJB2* | *GJB2* c.35delG (p.Gly12fs) | Homo | AR |
| 738 | Normal | *GJB2* | *GJB2* c.35delG (p.Gly12fs)  *GJB6-*D13S1830del | Het  Het | AR  AR |
| 745 | Normal | *GJB2* | *GJB2* c.35delG (p.Gly12fs) | Homo | AR |
| 757 | PVL | *GJB2* | *GJB2* c.35delG (p.Gly12fs)  *GJB6-*D13S1830del | Het  Het | AR  AR |
| 773 | Normal | *GJB2* | Causative mutation not available |  |  |
| 893 | PVL | *GJB2* | *GJB2* c.35delG (p.Gly12fs) | Homo | AR |
| 896 | Normal | *GJB2* | *GJB2* c.250G>T (p.Val84Leu)  *GJB2* c.617A>G (p.Asn206Ser) | Het  Het | AR  AR |
| 898 | Normal | *GJB2* | *GJB2* c.35delG (p.Gly12fs)  *GJB2* c.269T>C (p.Leu90Pro) | Het  Het | AR  AR |
| 902 | Normal | *GJB2* | *GJB2* c.35delG (p.Gly12fs)  *GJB2* c.44A>C (p.Lys15Thr) | Het  Het | AR  AR |
| 715 | Normal | *GJB2* | *GJB2* c.35delG (p.Gly12fs) | Homo | AR |
| 853 | Normal | *MYO15A* | *MYO15A* c.6863C>T (p.Ser2288Leu)  *MYO15A* c.4091_4099del (p.Arg1364_Asp1366del) | Het  Het | AR |
| 876 | PVL | *MYO15A* | *MYO15A* c.7226delC (p.Pro2409fs) | Homo | AR |
| 817 | PVL | *MYO6* | *MYO6* c.2751dupA (p.Gln918Thrfs*24) | Homo | AD |
| 844 | PVL | *MYO6* | *MYO6* c.470A>G (p.Lys157Arg) | Het |  |
| 770 | PVL | *POU3F4* | *POU3F4* c.607_610delCAAA (p.Gln203fs) | Hemi | X-linked |
| 820 | PVL | *POU3F4* | large chrom X deletion including  POU3F4/ZNF711/CHM/POF1B (arr[GRCh37] Xq21.1q21.31(80429329_91122135)x0 mat) | Hemi | X-linked |
| 742 | Normal | *TMPRSS3* | *TMPRSS3* c.208del (p.His70Thrfs*19) | Homo | AR |
| 912 | PVL | *MYO15A* | *MYO15A* c.6378G>C (p.Glu2126Asp)  *MYO15A* c.8215G>C (p.Ala2739Pro) | Het  Het |  |
| 919 | Normal | *GJB2* | *GJB2* c.35delG (p.Gly12fs) | Homo | AR |
| 926 | PVL | *GJB2* | Causative mutation not available |  |  |
| 930 | PVL | *TMC1* | *TMC1* c.215_219dupGGAGG (p.Arg74GlyfsX4)  *TMC1* c.1769G>T (p.Gly590Val) | Het  Het | AR |
| 928 | Normal | *GJB2* | *GJB2* c.35delG (p.Gly12fs) | Homo | AR |

**Abbreviations**: AR = autosomal recessive; AD = autosomal dominant; PVL = peripheral vestibular loss

**Supplemental Table 1B: Causative mutations for patients with syndromic hearing loss (n=20)**

| ID | Vestibular Function | Hearing Loss Etiology | Causative Genetic Variants | Zygosity | Inheritance |
| --- | --- | --- | --- | --- | --- |
| 733 | PVL | CAPOS | *ATP1A3* c.2452G>A (p.Glu818Lys) | Het | AD |
| 819 | PVL | CHARGE | Causative mutation not available |  |  |
| 847 | PVL | CHARGE | *CHD7* c.4034G>A (p.Arg1435His) | Het | AD |
| 795 | Normal | Coffin-Siris | *SOX11* c.965_983del19 (p.His322ArgfsX34) | Het | AD |
| 734 | PVL | JLNS | *KCNQ1* c.568C>T (p.Arg190Trp)  *KCNQ1* c.1075 C>T (p.Gln359*) | Het  Het | AR  AR |
| 748 | Normal | Noonan | *PTPN11* c.836A>C (p.Tyr279Ser) | Het | AD |
| 858 | PVL | SCID/RD | *AK2* c.524G>A (p.Arg175Gln) | Homo | AR |
| 862 | Normal | SCID/RD | *AK2* c.524G>A (p.Arg175Gln) | Homo | AR |
| 884 | Normal | SCID/RD | *AK2* c.524G>A (p.Arg175Gln) | Homo | AR |
| 857 | PVL | T21 | - |  |  |
| 718 | PVL | Usher | *MYO7A* c.640G>A (p.Gly214Arg)  *MYO7A* c.5573T>C (p.Leu1858Pro) | Het  Het | AR  AR |
| 756 | PVL | Usher | *PCDH15* c.1997+1G>A | Homo | AR |
| 792 | Normal | Usher | *CDH23* c.9629_9632del (p.Ile3210Argfs*5)  *CDH23* c.6050-15G>A  *CDH23* c.9199-4 G>A | Het  Het  Het | AR |
| 755 | Normal | WS2 | *MITF* c.(441+1_442-1)_(*1_?)del | Het | AD |
| 760 | Normal | WS2 | *MITF* c.773_785dup (p.Asp263GlyfsX5) | Het | AD |
| 908 | Normal | WS1 | *PAX3* c.821G>A (p.Trp274Ter) | Het | AD |
| 915 | PVL | Perrault | Causative mutation not available |  |  |
| 484 | PVL | Usher | *MYO7A* c.496delG (p.Glu166fs)  *MYO7A* c.3327delC (p.His1109fs) | Het  Het | AR  AR |
| 933 | PVL | PBD | *PEX1* c.2528G>A (p.Gly843Asp)  *PEX1* c.2383C>T (p.Arg795Ter) | Het  Het | AR  AR |
| 935 | PVL | WS2 | *MITF* c.91C>T (p.Gln31*) | Het | AD |

**Abbreviations**: AD = autosomal dominant; AR = autosomal recessive; PVL = peripheral vestibular loss; JLNS = Jervell and Lange-Nielsen syndrome; SCID/RD = severe combined immunodeficiency/reticular dysgenesis; T21 = trisomy 21; WS1 = Waardenburg syndrome type 1; WS2 = Waardenburg syndrome type 2; PBD = peroxisome biogenesis disorder

Red text indicates either (1) a variant of uncertain significance (VUS) found in the same gene as a pathogenic variant with recessive inheritance, or (2) a VUS considered potentially contributory by the clinical team.
